# Clinical and immunological features of an APLAID patient caused by a novel mutation in *PLCG2*

**DOI:** 10.3389/fimmu.2023.1014150

**Published:** 2023-01-27

**Authors:** Qi Peng, Dong Luo, Yi Yang, Yinghua Zhu, Qingming Luo, Huan Chen, Dapeng Chen, Zhongjun Zhou, Xiaomei Lu

**Affiliations:** ^1^ Department of Genetic Medicine, Dongguan Children’s Hospital Affiliated to Guangdong Medical University, Dongguan, China; ^2^ Department of Medical and Molecular Genetics, Dongguan Institute of Pediatrics, Dongguan, China; ^3^ Department of Genetics, Key Laboratory for Children’s Genetics and Infectious Diseases of Dongguan, Dongguan, China; ^4^ Department of infectious diseases, Dongguan Children’s Hospital Affiliated to Guangdong Medical University, Dongguan, China; ^5^ Department of Gastroenterology, Guangzhou Women and Children′ s Medical Center, Guangzhou, China; ^6^ Compartive Medicine Department of Researching and Teaching, Dalian Medical University, Dalian, China; ^7^ Faculty of Medicine, School of Biomedical Sciences, The University of Hong Kong, Hong Kong, Hong Kong SAR, China

**Keywords:** autoinflammation disease, PLCG2, phospholipase Cγ2, APLAID, NGS

## Abstract

**Background:**

The APLAID syndrome is a rare primary immunodeficiency caused by gain-of-function mutations in the *PLCG2* gene. We present a 7-year-old APLAID patient who has recurrent blistering skin lesions, skin infections in the perineum, a rectal perineal fistula, and inflammatory bowel disease.

**Methods:**

To determine the genetic cause of our patient, WES and bioinformatics analysis were performed. Flow cytometry was used for phenotyping immune cell populations in peripheral blood. Cytokines released into plasma were analyzed using protein chip technology. The PBMCs of patient and a healthy child were subjected to single-cell RNA-sequencing analysis.

**Results:**

The patient carried a novel *de novo* missense mutation c.2534T>C in exon 24 of the *PLCG2* gene that causes a leucine to serine amino acid substitution (p.Leu845Ser). Bioinformatics analysis revealed that this mutation had a negative impact on the structure of the PLCγ2 protein, which is highly conserved in many other species. Immunophenotyping by flow cytometry revealed that in addition to the typical decrease in circulating memory B cells, the levels of myeloid dendritic cells (mDCs) in the children’s peripheral blood were significantly lower, as were the CD4^+^ effector T cells induced by their activation. Single-cell sequencing revealed that the proportion of different types of cells in the peripheral blood of the APLAID patient changed.

**Conclusions:**

We present the first case of APLAID with severely reduced myeloid dendritic cells carrying a novel *PLCG2* mutation, and conducted a comprehensive analysis of immunological features in the ALPAID patient, which has not been mentioned in previous reports. This study expands the spectrum of APLAID-associated immunophenotype and genotype. The detailed immune analyses in this patient may provide a basis for the development of targeted therapies for this severe autoinflammatory disease.

## Introduction

Autoinflammatory diseases (AIDs) are rare inherited disorders involving a dysfunction of the innate immune system caused by genetic causes, characterized by recurrent episodes of fever and sterile inflammation, most commonly in the skin, intestines, joints, and eyes (1). The extremely rare, dominantly inherited PLCγ2-associated antibody deficiency and immune dysregulation (PLAID, OMIM# 614468) (2, 3) and autoinflammation and PLCγ2-associated antibody deficiency and immune dysregulation(APLAID, OMIM# 614878) (4, 5) are rare monogenic AIDs combining humoral immune deficiency and aseptic inflammation caused by *PLCG2* gene mutations. Patients with APLAID have early-onset blistering skin lesions and recurrent infections due to humoral defects, especially recurrent respiratory infections are the most common symptoms. But unlike PLAID, they lack circulating autoantibodies and do not present with cold-induced urticarial (6, 7).

APLAID is caused by *de novo* or dominantly inherited mutations in the *PLCG2* gene. The *PLCG2* (Phospholipase C Gamma 2) gene (OMIM* 600220) is located on chromosome 16q23.3 and contains 33 exons that code for PLCγ2, a cytoplasmic signaling enzyme that participates in the regulation of hematopoietic cell development and function (8). Herein, we report a Chinese APLAID patient with a novel heterozygous missense mutation c.2534T>C in the *PLCG2* gene. A comprehensive analysis of immune cells in the patient’s peripheral blood revealed that, in addition to the reduction in circulating memory B cells, the level of myeloid dendritic cells (mDC) in the peripheral blood was significantly reduced, as were the CD4^+^ effector T cells induced by mDCs, which had not previously been reported.

## Materials and methods

### Study subject

A 7-year-old boy born to non-consanguineous healthy Chinese parents with widespread blistering skin lesions, inflammatory bowel disease, a rectal perineal fistula, and perineal skin infections was recruited for this study. All biological specimens used in this study were collected at a single visit and after the patient was treated only with thalidomide tablets for only a short period of time. This study was approved and performed according to the protocol of the Institutional Medical and Ethics Committee of Dongguan Children’s Hospital Affiliated to Guangdong Medical University.

### Whole-exome sequencing and bioinformatics analyses

Whole exome sequencing was performed by the Guangzhou Jiajian Medical Testing Co., Ltd (Guangzhou, China) after obtaining informed consent. The evolutionary conservation of mutation sites was analyzed using multiple sequence alignment. The functional effect of the novel mutation was predicted using Polyphen-2 (9) and MutationTaster (10) online tools.

The three-dimensional (3D) structures of wild-type and mutant proteins were predicted using the iterative threading assembly refinement (I-TASSER) server (https://zhanglab.ccmb.med.umich.edu/I-TASSER/) (11). Wild-type and mutant protein amino acid sequences were submitted to the online I-TASSER software, an online resource for automated protein structure prediction and structure-based function annotation. The involved algorithms have been evaluated rigorously in community-wide blind experiments and showed significant advantages in protein structure and function prediction compared to peer methods. The flowchart of the I-TASSER server pipeline consists of three steps: template identification, full-length structure assembly and structure-based function annotation. The I-TASSER system used C-score to evaluate the accuracy of the models, C-score was typically in the range of (−5, 2); a higher value signified a model with more confidence and vice-versa (11). The model with highest C-score was chosen. The effect of mutations on protein configurations was visualized using PyMOL Viewer. The protein structure was created with Illustrator for Biological Sequences (IBS) (http://ibs.biocuckoo.org/). The pathogenicity of the mutation site was annotated following the American College of Medical Genetics and Genomics guidelines (ACMG) (12).

### Immune function evaluation

Serum immunoglobulins (IgA, IgM and IgG antibody) levels were assessed *via* chemiluminescence immunoassay. A comprehensive analysis of the proportions and absolute numbers of immune cells in peripheral blood using flow cytometry was conducted by Guangzhou KingMed Centre for Clinical Laboratory Co., Ltd (Guangzhou, China). RayBio^®^ C-Series Human Inflammation Antibody Array (AAH-INF-3-2) for the semi-quantitative detection of 40 human cytokines, was employed for automatically detect cytokines from plasma of the patient and a healthy child by Guangzhou RayBiotech Biotechnology Co., Ltd. To explore the differentially plasma cytokines between the patient and heathy child, cytokines with 1.2-fold change differences were set as the screening criteria. Gene Ontology (GO) enrichment and KEGG analysis were performed to explore the possible biological function of the differentially expressed cytokines.

### Single-cell RNA sequencing

Single-cell suspensions from our patient and a healthy child were prepared referred to the method as described (13). After Drop-seq droplet collection, complementary DNA was synthesized and amplified, and then its quality was evaluated. Sequencing using an Illumina Novaseq6000 System and data processing were performed by CapitalBio Technology (Beijing, China).

## Results

### A novel *PLCG2* mutation was identified in the patient

**Figure 1:**
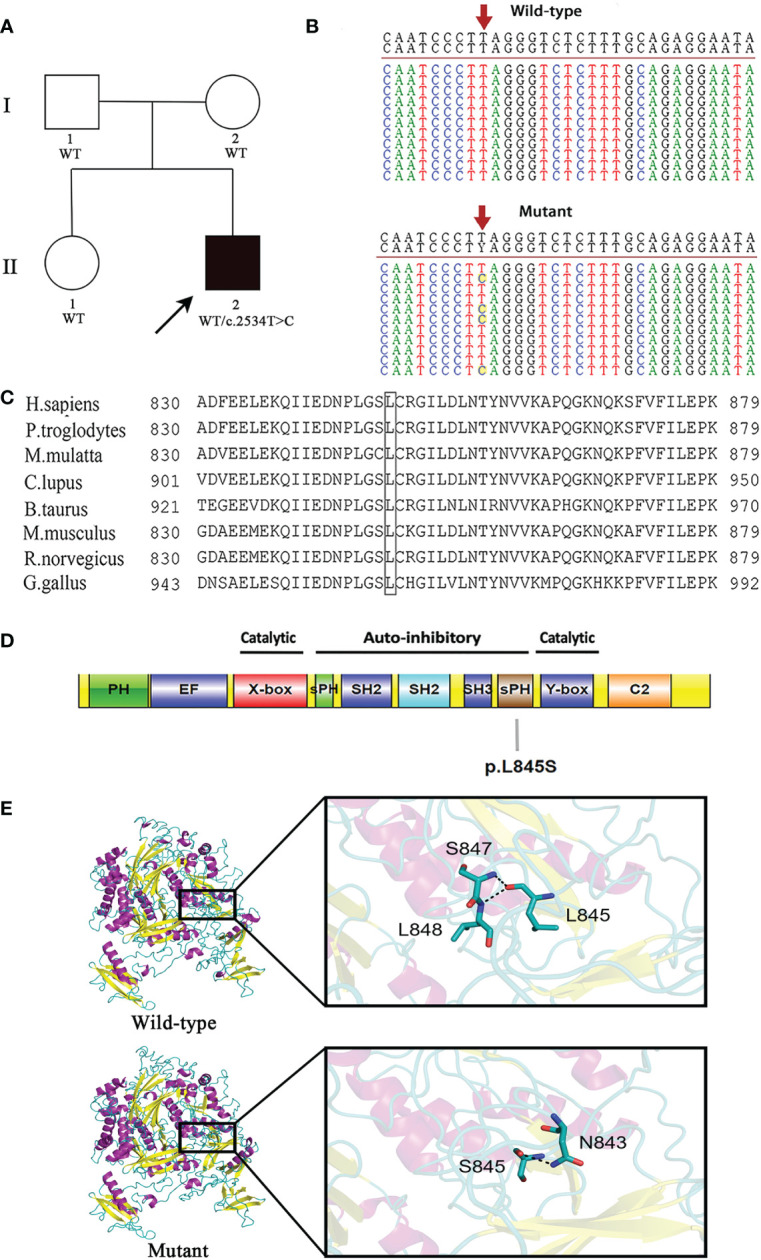
A novel *de novo* heterozygous mutation is identified in *PLCG2*. **(A)** Pedigree of the affected family. Circles, females; squares, males; filled symbol, affected subject; open symbols, unaffected family members. The proband is indicated by an arrow. **(B)** DNA sequencing of wild-type (WT) and mutant (MUT) *PLCG2*. The red arrow indicates the position of the c.2534T>C variant in *PLCG2*. **(C)** The protein alignment shows conservation across eight species. The mutation c.2534T>C occurred at evolutionarily conserved amino acids. **(D)** Schematic representation of the protein domains of PLCγ2. The mutation identified in the current report was showed. **(E)** Protein structure modeling of wild-type and mutated PLCG2.

This patient is not a carrier for rare or common pathogenic variants associated with IBD. A novel heterozygous pathogenic variant c.2534T>C in exon 24 of the *PLCG2* gene was identified in the proband. It was absent from both parents and his healthy sister, indicating that this mutation was *de novo* in the patient (**Figures 1A, B**).

The c.2534T>C mutation is not present in the ExAC, dbSNP version 147, HGMD, 1000 genomes project, or Clinvar databases, and it has not been reported previously. A predicted amino acid substitution of leucine to serine resulted from the novel mutation. Multiple sequence alignment was performed for eight diverse vertebral species, and the results indicated that codon 845 is highly conserved during evolution and has functional importance (**Figure 1C** **)**. Moreover, it was predicted to be damaging by Polyphen-2 and disease causing by MutationTaster.

As with the majority of other PLC isoforms, PLCG2 has several core domains, which includes an N-terminal PH domain, two pairs of EF hands, a TIM catalytic cylinder made up of the X- and Y-boxes, and a C2 domain. This core is lined with a special collection of regulatory domains found only in PLCG isoenzymes, such as a split pleckstrin homology (sPH) domain, two SH2 domains, and an SH3 domain. The amino acid residue Leu845 affected by this novel mutation is situated in the sPH domain of the PLCγ2 protein (**Figure 1D**).

The modeling with I-TASSER gave five models for each protein. The best structure with high confidence score was collected and used for further investigations (wild-type PLCγ2 protein model with C‐score = -0.15 and estimated TM-score = 0.69 ± 0.12, mutant PLCγ2 protein model with C‐score = -0.34 and estimated TM-score = 0.67 ± 0.13). The structure of the mutant PLCγ2 protein differs greatly from that of the normal protein, as illustrated in **Figure 1E**. In the structure of the wild protein, the carbonyl oxygen of L845 forms hydrogen bonding interactions with the backbone amides of S847 and L848. However, in the structure of the L845S mutant, the O atom of the side chain of S845 donates a hydrogen bond to the N atom of the side chain from N843, which might influence the interactions of S845 with S847 and L848. We speculated that the difference of the protein structure might have a significant impact on the protein functions.

This variant may be categorized as likely pathogenic in accordance with ACMG guidelines. The specifics are as follows: 1) *de novo* (both paternity and maternity confirmed) in a patient with a negative family history of the disease (PS2); 2) absence in the general population (PM2); 3) pathogenic gain-of-function variants at the same amino acid residue, the somatic mutations c.2535A>C (p.Leu845Phe) and c.2535A>T (p.Leu845Phe), were identified in patients with chronic lymphocytic leukemia who were resistant to treatment with Bruton tyrosine kinase inhibitor, ibrutinib (PM5) (14); 4) this variant is expected to have a detrimental effect on protein function by multiple lines of computational algorithms (PP3). This mutation can be found in ClinVar database (https://www.ncbi.nlm.nih.gov.clinvar/) using accession number SCV002540197.

### Clinical symptoms and laboratory data

**Figure 2:**
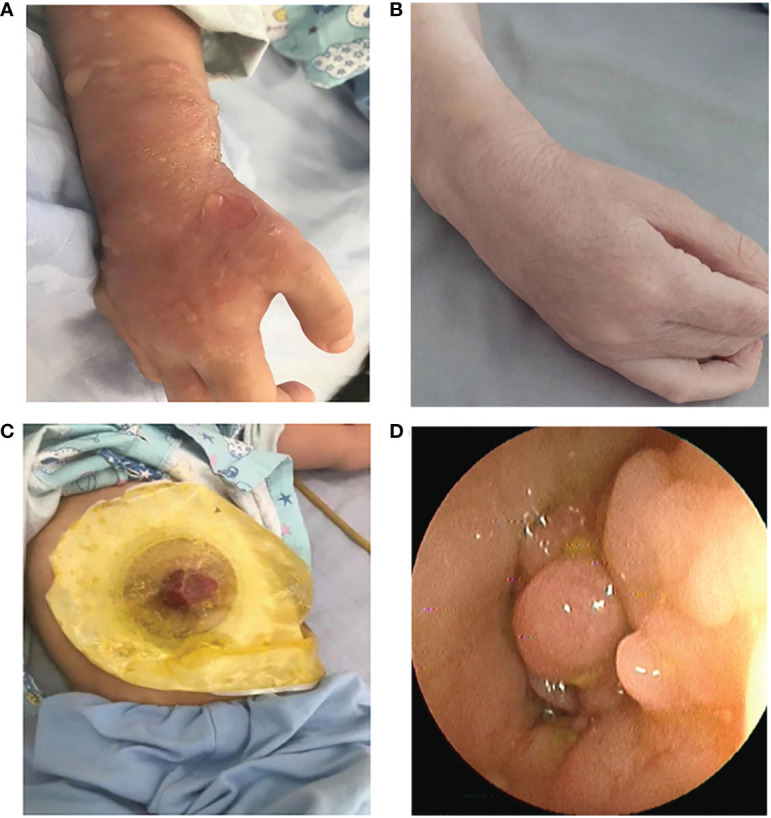
**(A)** Recurrent eruptions of vesiculopustular lesions. **(B)** The skin lesions resolved after antibiotic treatments, and he currently has loose skin. **(C)** Severe inflammatory colitis, necessitating a colostomy. **(D)** Representative images of colonic lesions in the patient.

The proband have extensive vesicular skin lesions and secondary infection (**Figure 2A**). The skin lesions resolved after antibiotic treatments, and he currently has loose skin (**Figure 2B**).In this child, unlike most previously reported cases, respiratory infections are uncommon. However, the patient had severe infections involving the scrotum and perianal area, and suffered from severe inflammatory colitis, necessitating a colostomy (**Figure 2C**). Colonoscopy revealed that the child’s intestinal mucosa was congested and edematous, with multiple finger-like or mass-like inflammatory proliferative lesions that were brittle and easily bled (**Figure 2D**). Thalidomide, a TNF inhibitor, had been administered to the patient prior to the time of sampling, but it was only partially effective with a slight decrease in the discharge surrounding the anus for a short period of time, and stopped soon. After that, infliximab injection for four courses of therapy, mesalazine, and compound glutamine granules were used, but none of them showed positive results; in fact, the patient even experienced symptoms of diarrhea. Currently, patient relies on high doses of the hormone meprednisone (24 mg/d) to stop the condition from getting worse.

His white blood cell count was 14.56×10^9^/L (normal range: 4.00-10.00×10^9^/L) with 1.15×10^9^/L monocytes (normal range:0.10-0.60×10^9^/L); hemoglobin was 86g/L (normal range: 120-140 g/L); and C-reactive protein was 96.26 mg/L (normal ≤ 6).As show in **Table 1**, his serum IgA level was normal, but he had lower IgG and IgM levels: IgG (4.97g/L; reference range [RR]: 5-10.6) and IgM (0.10g/L; RR: 0.44-1.44). Results from flow cytometry revealed B cell lymphopenia, with a near total absence of marginal zone B cells, memory B cells, and class-switched B cells; the remaining B cells were mostly immature (transitional B cells and Naive B cells). The number of natural killer (NK) cells was within the normal range. In terms of T lymphocytes, the total number was normal, but the numbers of functional CD4+ effector T cells and regulatory T cells (Tregs) were significantly lower. It showed a significant increase in monocytes, primarily due to a significant increase in the number of classical monocytes, which was consistent with the results of routine blood tests. His total number of dendritic cells is reduced, with myeloid dendritic cells being the most severely affected.

**Table 1:** Results of the immunological investigations.

Immunological Investigation	Results	Reference Values	Unit
Immunoglobulins			
IgA	0.60	0.34-1.38	g/L
IgG	**4.97 ↓**	5.0-10.6	g/L
IgM	**0.10 ↓**	0.44-1.44	g/L
Flow cytometry Immunophenotyping			
B lymphocytes	24.00	51.00-728.00	cells/μl
Transitional B lymphocytes	1.00	0.00-20.00	cells/μl
Naive B cells	22.00	5.00-401.00	cells/μl
Marginal zone B cells	**0.00 ↓**	1.00-84.00	cells/μl
Memory B cells	**0.00 ↓**	3.00-80.00	cells/μl
Class switched B cells	**0.00 ↓**	1.00-53.00	cells/μl
T lymphocyte	1992.00	270.00-2586.00	cells/μl
Naive CD4^+^T cells	192.00	4.00-1079.00	cells/μl
Central memory CD4^+^ T cells	**910.00 ↑**	70.00-671.00	cells/μl
CD4^+^ effector T cells	**2.00 ↓**	23.00-301.00	cells/μl
CD4^+^ effector memory T cells	44.00	0.00-141.00	cells/μl
Naive CD8^+^T cells	257.00	18.00-355.00	cells/μl
Central memory CD8^+^ T cells	**218.00↑**	7.00-206.00	cells/μl
CD8^+^ effector T cells	**7.00↓**	8.00-635.00	cells/μl
CD8+ effector memory T cells	191.00	13.00-457.00	cells/μl
Regulatory T cells (Tregs)	**24.00 ↓**	28.00-142.00	cells/μl
Dendritic cell (DC)	**10.00 ↓**	20.00-121.00	cells/μl
Plasmacytoid dendritic cells (pDC)	6.00	1.00-21.00	cells/μl
Myeloid dendritic cells (mDC)	**1.00 ↓**	10.00-107.00	cells/μl
CD16^+^ dendritic cells (CD16^+^ DC)	**0.00↓**	5.00-95.00	cells/μl
Monocyte	**1036.00↑**	144.00-702.00	cells/μl
Classical monocytes	**958.00↑**	114.00-589.00	cells/μl
Intermediate monocytes	44.00	7.00-70.00	cells/μl
Pro-inflammatory Monocyte	25.00	7.00-86.00	cells/μl
NK cell	339.00	53.00-569.00	cells/μl
Mature NK cells	317.00	48.00-557.00	cells/μl
Immature NK cells	22.00	2.00-33.00	cells/μl

↑ and ↓ arrows denote increased and decreased levels, respectively. The index value that is outside of the reference range is shown in bold.

### Differentially plasma cytokine screening

**Figure 3:**
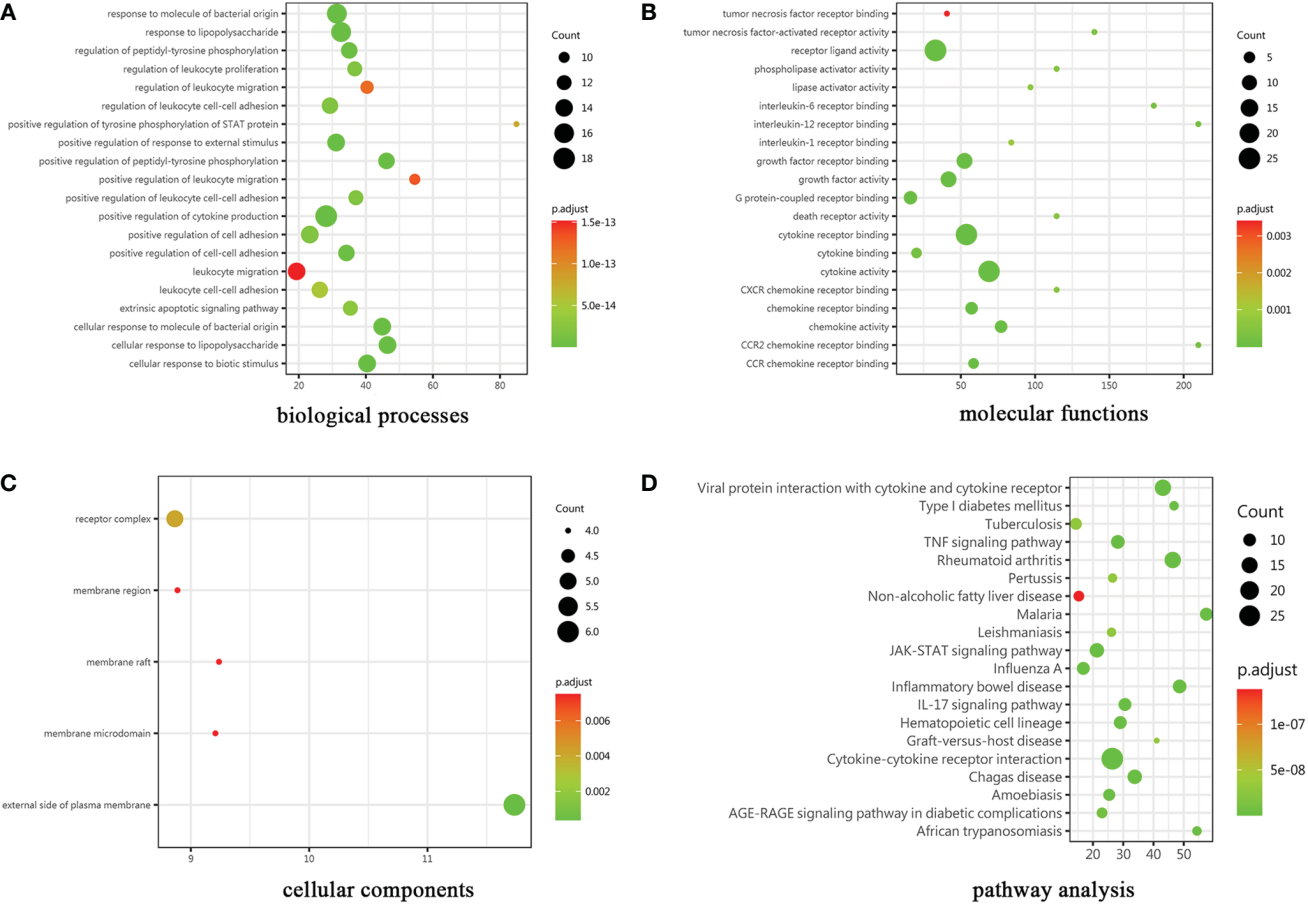
Results of GO and KEGG pathway analysis. The gene ontology categories were **(A)** biological processes, **(B)** molecular functions and **(C)** cellular components. **(D)**KEGG: Kyoto Encyclopedia of Genes and Genomes.

The cytokines with ratio values of less than 0.83-fold or more than 1.2-fold or (absolute logFC>0.263) were regarded as differentially expressed proteins (DEPs). 30 DEPs were found in the patient compared to control, 24 (80%) of which were down-regulated, and 6 (20%) were up-regulated. Complete lists of DEPs that have considerably changed can be seen in **Supplementary Data 1**. The results of the GO enrichment analysis are provided in **Figure 3**. The color gradient in the Figure, which runs from red to green, shows the size of the p value; the closer to green, the smaller the p value; and the higher the significant level of enrichment of related GO annotation. Many terms in the biological processes (BP) category are related to positive regulation of cytokine production and response to molecule of bacterial origin (**Figure 3A**). Cytokine activity, cytokine receptor binding, and receptor ligand activity are the main enrichment of molecular functions (MF) (**Figure 3B**). These genes were primarily localized in the plasma membrane’s outer layer, according to cellular components (CC) analysis (**Figure 3C**). In addition, many distinct KEGG signaling pathways were identified such as JAK-STAT signaling pathway, viral protein interaction with cytokine and cytokine receptor signaling pathway, and TNF signaling pathway etc (p<0.001) (**Figure 3D**). The cytokine-cytokine receptor interaction pathway had the most enriched genes and was the most significantly enriched functional category. Interestingly, a wide variety of cytokines such as IL-13, IL-10, and TGFβ1 have been demonstrated to participate in the innate and adaptive immune response to inflammatory bowel disease (IBD).

### A single-cell transcription atlas of PBMC in the APLAID patient

**Figure 4:**
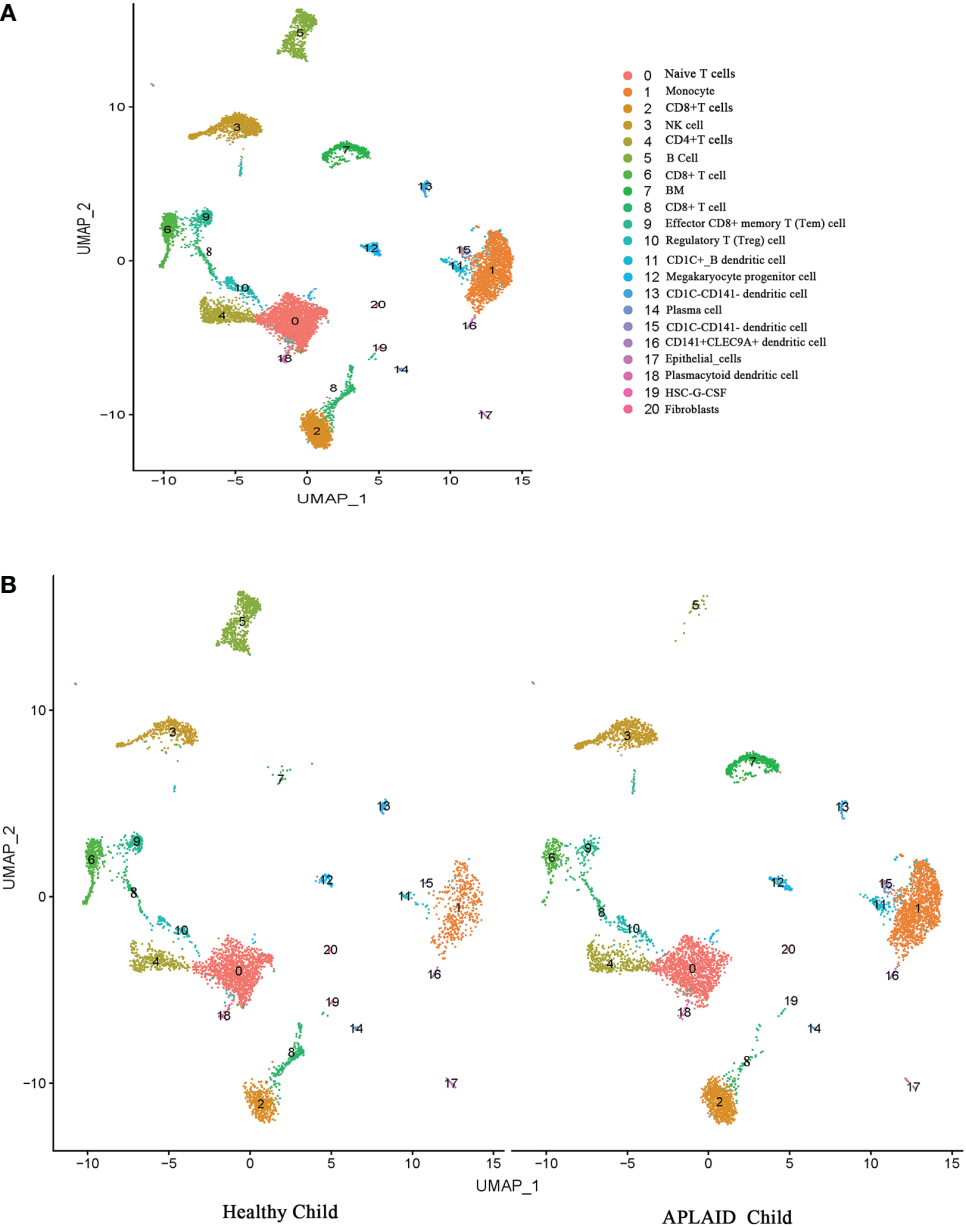
Cell types identified in peripheral blood mononuclear cells (PBMC) by uniform manifold approximation and projection (UMAP). Each dot represents a cell, which is colored according to cell type. **(A)** Merged cell clusters in two samples. **(B)** 21 cell clusters in healthy child (left) and APLAID child (right) PBMC samples, respectively.

Transcriptomes of 6583 and 5306 single cells from the APLAID patient and healthy child samples were collected after rigorous quality control and filtering by several criteria, with a total detected gene of 19,984 and 19,763, respectively (**Supplemental Table S2**). We employed the UMAP algorithm to cluster cells with comparable expression patterns *via* dimension reduction based on principal component analysis (PCA). Both samples were classified into 15 clusters. After that, two single-cell datasets were combined to allow for a thorough comparison of the APLAID patient and healthy child. **Figure 4A** shows 21 cell clusters that can be recognized based on the expression of gene markers (marker genes correspond to the CellMarker Database). We discovered a substantial difference in immune-related cell clusters by comparing the composition of cell subpopulations in APLAID and healthy child. The cluster 5 B cells were significantly decreased and cluster 1 monocytes were increased (**Figure 4B**). As for the difference of cluster 7 is maybe due to the fact that the patient has anemia due to thalassemia, so there is a certain amount of erythrocytoblasts in the peripheral blood. Overall, the above results show that there were differences in immune cell subpopulation in ALPAID patient.

## Discussion

In this study, we present a case of a 7-year-old boy with APLAID who had blistering skin lesions, perineum skin infections, a rectal perineal fistula, and severe inflammatory bowel disease. A *de novo* heterozygous missense mutation c.2534T>C (p.Leu845Ser) of *PLCG2* gene was found in the patient. The mutation has only been reported in this study, as far as we are aware.

*PLCG2* p.Leu845 is highly conserved indicating that it plays an important role in cells and that any disruption could lead to a malfunctioning protein. A polar amino acid (Serine) replaces a nonpolar amino acid (Leucine) in the novel missense mutation, which may have a substantial effect on protein structure. A negative effect on the 3D structure of the PLCG2 protein is caused by this mutation according to the bioinformatic analysis. The predicted consequences of our mutation using PolyPhen2 and SIFT were in agreement with the bioinformatics study. This mutation is in the PLCG2 split PH domain (sPH), which is part of the autoinhibitory component of the PLCG2 gene and normally couples the enzymatic activity of PLCG2 to upstream pathways. Thus, in our case, the novel variant might result in the release of autoinhibition, resulting in activation of PLCγ2. APLAID is an extremely rare monogenic auto inflammatory disorder, with a difficult diagnosis process in children.

A neonatal vesiculo-pustular eruption that resolves on its own within the first few weeks of life, recurrent episodes of discrete erythematous papules that develop into plaques with grouped vesicles and pustules, and sizable hemorrhagic blisters that may ulcerate are all shared skin features in APLAID patients (4, 15). Common systemic, non-cutaneous APLAID symptoms include enterocolitis with recurring abdominal discomfort and bloody diarrhea, interstitial pneumonitis, sinopulmonary infections, arthralgia, eye inflammation (5, 16). However, our patient does not have recurrent lung infections, eye inflammation, and joint symptoms, but are mainly manifested by skin breakage and severe inflammatory colitis.

It is well known that PLCγ2 is necessary for B cell receptor (BCR) signaling, which is crucial for B cell development and maturation (17). APLAID patients have been previously described had varied degrees of B cell lymphopenia and antibody deficiency, but no obvious impairment of T or NK functions (4, 6, 16). Our patient also displayed a notable reduction in B lymphocytes, which was consistent with previous reports. The difference noted in our patient was low dendritic cell counts, mainly myeloid dendritic cells, which were not mentioned in previous reports. Such cells may not have been analyzed. It is worth mentioning that the single-cell sequencing results did not show obvious abnormalities in DC cells, which may be because the number of cells captured by single-cell sequencing is far less than the number of cells analyzed by flow cytometry, and the number of DC cells in humans is very low, so the single-cell sequencing results cannot fully reflect the composition of DC cells.

PLCγ2 is a crucial signaling protein activated downstream by a variety of cell surface receptors that contain an intracellular immunoreceptor tyrosine-based activation motif. It is important to myeloid cells including monocytes, macrophages, NK cells, dendritic cells (DCs), and mast cells often because of its utility in promoting downstream signaling after FcR engagement (18).In this patient carrying PLCG2 c.2534T>C mutation, mDC cells were significantly reduced. Dendritic cells (DCs) play a key role in triggering protective immune responses against infections. They act as a bridge between innate and adaptive immunity, and are able to present processed peptides derived from diverse antigens, initiating and regulating the adaptive immune response by activating lymphocytes (19, 20). DCs are classified into two main subtypes: plasmacytoid DCs (pDCs) and myeloid DCs (mDCs), which are specialized in recognizing different pathogen-associated molecular patterns (PAMPs) (21). As a result, mDCs and pDCs can effectively trigger CD4^+^ and CD8^+^ T cell responses against distinct pathogen types. However, the number of CD4^+^ effector T cells produced by mDC cells was dramatically decreased in our patients, while the number of CD8^+^ effector cells did not change significantly. The precise mechanism underlying this phenomenon has to be further researched.In this patient, a number of cytokines secreted by DC cells such as IL-10, IL-15, TNF-α, etc. are also reduced. For example, immunomodulatory cytokine IL-10 is known to modulate the immune response in the gut, and IBD has been associated with impaired signaling in the IL-10 pathway (22, 23). Given our patient has severe IBD, it’s tempting to assume that reduced related cytokines production causes immunological dysregulation and is to blame for the intestinal inflammation in our patient. In addition, the patient’s monocyte count was significantly increased and his regulatory T cells significantly reduced, which was not mentioned in the previously reported cases. Therefore, the underlying mechanisms need to be further investigated.

In summary, APLAID is an autoinflammatory disease difficult to diagnose and treat due to its rarity and heterogeneous clinical manifestations. We identified a novel mutation in the sPH domain of PLCG2 in a Chinese APLAID patient with low dendritic cell counts and high monocyte counts that may expand the genotypes and immune phenotypes of APLAID.

## Data availability statement

The data presented in the study are deposited in Figshare, https://doi.org/10.6084/m9.figshare.21844845.v1, and ClinVar, accession number SCV002540197.

## Ethics statement

The studies involving human participants were reviewed and approved by the Institutional Medical and Ethics Committee of Dongguan Children’s Hospital Affiliated with Guangdong Medical University. Written informed consent to participate in this study was provided by the participants’ legal guardian/next of kin. Written informed consent was obtained from the minor(s)’ legal guardian/next of kin for the publication of any potentially identifiable images or data included in this article.

## Author contributions

XL conceived, designed and guided the study, and revised the manuscript critically. QP, DL, YY performed the experiments. HC, YZ,QL collected and analyzed the data. QP wrote the manuscript. DC and ZZ checked and revised the manuscript. All authors contributed to the article and approved the submitted version.
